# Chief medical officers in the United Kingdom: maintaining ‘independence’ inside government

**DOI:** 10.1093/pubmed/fdae278

**Published:** 2024-10-30

**Authors:** Katherine E Smith, Anna Macintyre, Margaret MacAulay, Patrick Fafard

**Affiliations:** Centre for Health Policy, University of Strathclyde, Glasgow G4 0LT, UK; Centre for Health Policy, University of Strathclyde, Glasgow G4 0LT, UK; Global Strategy Lab, York University and University of Ottawa, 4700 Keele Street, Toronto, M3J 1P3, Canada; Global Strategy Lab, York University and University of Ottawa, 4700 Keele Street, Toronto, M3J 1P3, Canada; Faculties of Social Sciences and Medicine, University of Ottawa, 75 Laurier Ave E, Ottawa, ON K1N 6N5, Canada

**Keywords:** chief medical officer, COVID-19, health policy, public health, scientific advice, United Kingdom

## Abstract

**Background:**

The Chief Medical Officer (CMO), one of the UK’s most senior public health leadership roles, was crucial in supporting policymakers in responding to COVID-19. Yet, there exist only a handful of (largely historical) accounts of the role in England. This article is the first to empirically examine how the scope, focus and boundaries of the CMO role vary over time across the four UK nations, including during public health emergencies.

**Methods:**

We undertook semi-structured interviews with 10 current and former CMOs/Deputy CMOs in the four UK nations and analysed relevant documents.

**Findings:**

The CMO role is not clearly defined in contemporary UK legislation and is instead shaped by iterative policies, incumbent preferences, and organizational needs, leading to variation over time and between nations. Nonetheless, most participants framed the role as primarily providing ‘independent’ advice to government despite being senior civil servants who, in communicating with the public, sometimes speak ‘on behalf’ of government.

**Conclusions:**

The flexibility of UK CMO roles allows for responsive adaption but poses risks for how well these roles are understood. A potential tension between providing ‘independent’ policy advice and a need to publicly communicate government policies and guidelines may be exacerbated in emergency contexts.

## Introduction

The COVID-19 pandemic brought unprecedented visibility and attention to chief medical officers (CMOs).^1^^,^^2^ As ‘the nation’s doctor’, the CMO is ‘the most senior government adviser on health matters’,^2^ intersecting the state, medicine and the public.^3^ Yet few publications examine the role.^1^^,^^3–8^ An analysis of CMOs in the European Union found the role varies substantially,^5^ while Canadian analysis of the equivalent role consistently identified three main functions: advisor, communicator and manager.^4^^,^^9^ Given limited contemporary analysis of CMO roles in the UK,^5^^,^^10^^,^^11^ our design was informed by this analysis.

During the COVID-19 pandemic, secondary sources were used to summarise CMO roles across the UK^12^^,^^13^ but centring on England, an important limitation, given concerns about the coordination across the UK’s devolved scientific advisory systems.^2^^,^^11^ Drawing on a combination of documentary analysis and interview data, we address two research questions:

1. What are the scope and boundaries of the CMO role across the devolved UK and how does this vary between the four nations (England, Scotland, Wales and Northern Ireland)?
2. How do CMO roles in the devolved UK adapt to respond to public health emergencies?

**Fig. 1:**
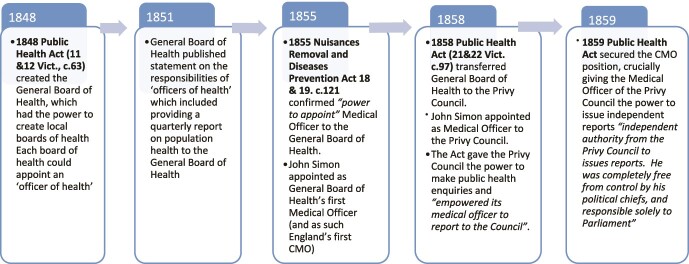
Key historical developments in the evolution of the CMO role in England. (Sources: 3,12,14–16)

We situate our findings in a recent political science analysis of the ‘politics of experts’,^10^ which conceives of UK CMOs as ‘core insiders’ who have a direct link to ministers but struggle to manage the conflicts between the ‘rules of the game’ governing public servants (i.e. confidentiality and low-profile) and those shaping science (transparency and visibility).

## Methods

We analysed relevant literature, documents and legislation (searching SCOPUS, government webpages and Google for ‘CMO’ and the four UK nations January 2021–April 2024) to identify the role’s statutory and non-statutory elements (Supplementary File).

Next, we undertook semi-structured interviews with 10 individuals with experience in the CMO or Deputy CMO (DCMO) roles, ensuring representation across all four UK nations. Participants were approached via emails outlining the study’s purpose. Interviews were conducted and recorded virtually (via Zoom/Teams) April–December 2021 with two (or more) members of the research team, allowing one to ask topic guide questions (informed by documentary and Canadian analysis^4^^,^^9^; see Supplementary file), while another developed follow-up questions. Audio recordings were professionally transcribed, anonymised and checked for accuracy.

We conducted a qualitative analysis of the interview transcripts in NVivo (R1), using a deductive coding framework informed by our research questions and documentary analysis (supplemented with inductive codes relating to external factors interviewees described as shaping the CMO role). We coded transcripts via an iterative process: MM read all transcripts to identify additional codes not covered by the initial framework; AM then reviewed MM's coding of two transcripts before MM finalised the framework and coded remaining transcripts.

We recruited individuals who held the CMO/DCMO role during the COVID-19 pandemic or who held such roles in the past 25 years (all but one in the past decade). Our interview data, therefore, combine direct insights about how the role adapted during COVID-19, with more reflective accounts of those at a distance from the role, several of whom dealt with previous public health emergencies.

The research was reviewed and approved by the University of Ottawa's Research Ethics Board (S-12-20-6249) and submitted to the University of Strathclyde's Research Ethics Committee for secondary approval. Reflecting the consent provided and ensuring we focus on the role; we use numerical IDs (rather than interviewee names).

**Table 1:** The four consistent dimensions of the CMO role that interviewees across the devolved UK referred to

*CMO role dimension*	*Illustrative extracts from interviewees*
D1. Government advice, bringing scientific and professional perspectives	‘I supported the Minister in scrutiny hearings and looking at the evidence and bringing experts to advise on what the issues were and what the science was telling us.’ (006) ‘We provide the public health and the scientific and the medical advice to our political colleagues, ministers, and it is the ministers who make the decisions.’ Also described key part of role being to provide advice and guidance based ‘on the best determination that we have in terms of the evidence.’ (008) Others described helping to ‘provide clinical and public health advice’ (005), ‘build up the evidence’(004), ‘know what the evidence is and what the evidence isn’t’ (004) and ‘provide an evidence base for decisions.’ (007)
D2. Public communication	‘That is part of the role, you are the public’s doctor […] so that would be […] explaining and communicating effectively.’ (006)
D3. Public health advocacy	‘I was always reminding ministers as to what the consequences, the unintended consequences of […] what they wanted to do would be, especially if it affected some of the issues that you were interested in yourself. So that was a bit about how the CMO’s own particular agenda began to shape the kind of dialogue that you had.’ (001) ‘Myself and [another incumbent] got together, and over a period interviewed clinicians, the public, other ministers, other services, and brought together a report, which was, I think, quite a successful one. It showed the range of people involved, and the ways of gathering information, so that it was strongly supported. At the end of the project, people said, “Yes, let’s do this.” That was part of the objective of the whole project.’ (004) ‘Sometimes we think very traditionally that the person who is the advocate is the person who’s the loudest, who’s waving a flag for various causes. And that is one way. I think there are other really important ways; building the relationships, building the partnerships, making sure that the advice we’re giving and the work we’re doing is properly grounded in relationships with the public and with the people we serve.’ (008)
D4. Line management (of other civil servants)	‘The line management people I had responsibility for were the folks working within the department of health, […] the doctors, the medical officers and so on working in department of health management.’ (001).

## Results

### Historical evolution of the CMO role

Our analysis focuses on the CMO role in contemporary times, but Fig. 1 provides an annotated timeline of the early evolution of the CMO role in England.^3^^,^^12^^,^^14–16^ Three features are worth noting. First, the statutory basis for the CMO role remains tied to mid-19^th^ century English public health legislation.^3^^,^^12^^,^^15^ Second, the statutory basis for the CMO’s duty to report first appeared in 1851^16^ and, crucially, the Public Health Act in 1859 gave the Medical Officer of the Privy Council the ‘independent authority’ to issue reports to Parliament.^3^ This is core to understanding the relative independence of the CMO role. Third, the role continued to evolve with subsequent legislation and each new incumbent, leading Sheard and Donaldson to quip, ‘The post of CMO has perfectly fitted the old adage “a job is what you make it”.’^3^

Our documentary analysis suggests the statutory basis of contemporary CMO roles is far from clear in the UK (Supplementary File); when asked, few interviewees reported engaging with the role’s legislative basis. Most recent public health acts make no mention of CMOs, with some secondary legislation briefly mentioning them as those who ‘notify’ or are ‘notified by’ others regarding infectious disease outbreaks (Northern Ireland is an outlier here, since secondary legislation specifies the CMO must notify GPs and other health professionals regarding an influenza outbreak^17^). In contrast, the CMO appears in many other pieces of ‘non-public health’ legislation, including on abortion, social security, and general public administration, usually in terms of the role’s advisory function. In short, the CMO office is one that Ministers and officers have a duty to consult with or notify, yet there is little specification of the statutory responsibilities of office-holders themselves. The exception is emergency legislation on the COVID-19 pandemic (e.g. the Coronavirus Act 2020), which specifies the role’s advisory function, though it does not make explicit any other CMO responsibilities.

### CMOs in the contemporary UK

**Fig. 2:**
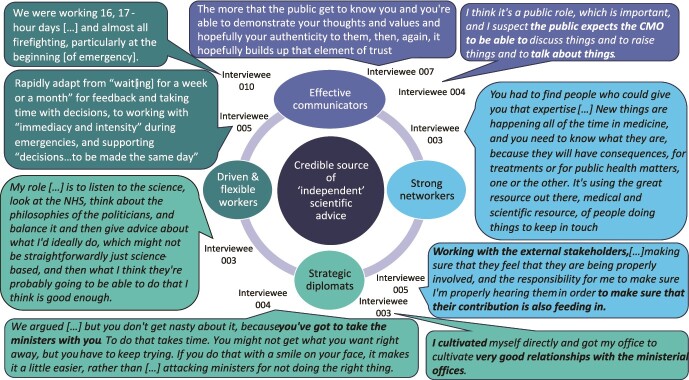
The attributes that interviewees suggested individuals require to succeed in a UK CMO role.

In interviews, CMOs describe four role dimensions (Table 1): scientific policy advisor, public communicator (of government decisions or scientific findings), health advocate (usually undertaken internally) and line manager of other civil servants. However, the balance varies, reflecting individual preferences and contextual (e.g. organisational) factors. Each dimension is potentially substantial and can expand rapidly (e.g. during public health emergencies) but different dimensions do not always align. To successfully navigate all this, interviewees’ accounts suggest that CMOs in the UK require five attributes (Fig. 2). Of these, interviewees consistently emphasised the importance of credibility, often linking this to being recognised as ‘independent’ scientific advisors:

‘Everyone knows I'm independent. There's no way any minister could make me do something that went against my values or the evidence.’ (003)

‘I think that independence is really important.’ (006)

‘I couldn't be more sort of adamant about the importance of being independent of government, and that's a very important part written into that CMO role.’ (010)

This emphasis is interesting given UK CMOs are, technically, senior civil servants who are bound by the same protocols as others.^10^^,^^18^ When asked about potential tensions between being independent and a senior civil servant, interviewees suggested they found it unproblematic (a contrast with their Canadian equivalents^9^). However, interview data also suggest that each dimension must be carefully managed to maintain this perceived independence.

Four tensions are evident. The first relates to CMOs’ dual status as^1^: ‘core insiders’,^10^ bound by civil service rules emphasising low visibility and confidentiality; and^2^ ‘independent’ scientific advisors with the unique authority to independently report to the public.^13^ While this furnishes CMOs with greater opportunity to publicly criticise government decisions, interviewees emphasised their ability to speak openly to ministers behind closed doors, in keeping with their ‘core insider’ status.^10^ Participants also generally accepted that their advice would often be balanced with ‘the political reality of the world’ (002).

However, not speaking out against a government position, ignoring strong scientific advice, or a clear consensus among health professionals, compromises the perceived independence of CMOs.^19^ Reflecting this, several interviewees recalled situations in which they either had spoken out or considered it. For example:

‘I think, only once, the four CMOs, […] objected to [a proposal]. I think we would all have taken serious action if it hadn't changed. It changed, and we didn't take any action, but I think there are times when you've got to go right up to the wall.’ (004)

It was in describing these situations, participants underlined the importance of strategic diplomacy (Fig. 2). This involved utilising intra-UK CMO relationships or internal policy networks to either prevent a need to publicly challenge government or limit relational damage on the rare occasions it happened. Interviewees consistently came across as skilled diplomats carefully balanced public and policy advisory role dimensions.

The second tension arises from the public communication role. In non-emergency times, interviewees suggested public communications focused on CMO reports or summarising scientific evidence, which did not require speaking ‘on behalf’ of the government. Interestingly, while two interviewees maintained that CMOs were always independent, other interviewees described how the CMO role did require speaking on behalf of the government, notably during public health emergencies:

‘Sometimes [we are speaking] on behalf of the science and the evidence and what we're advising people to do, and [sometimes] we are speaking on behalf of the department…I think we can be doing each at different times.’ (008).

‘I did quite a lot of media during that [public emergency] and yes, of course, I was speaking on behalf of government.’ (002)

As several noted, speaking *on behalf of* the government appeared to conflict with the perceived independence of the CMO role.

The third tension arises from advocacy. Here, interviewees described using their ‘soft and quiet influence’ (007) as ‘core insiders’^10^ to promote topics of personal interest and/or to protect marginalised communities. For example:

‘A lot of what we have to do is probably a little bit more covert, and under the radar. So, it isn't always possible for us to speak up on certain things […] It isn't that we're not doing it […] but we have to work, and operate, within the paradigm that is appropriate to us.’ (009)

Some described promoting issues via alliance-building. One interviewee suggested that while advocates are traditionally imagined as loudly ‘waving a flag for various causes’, other important mechanisms involve ‘building the relationships, building the partnerships, making sure that the advice we’re giving and the work we’re doing is properly grounded in relationships with the public.’ (008).

However, recalling the first tension, CMOs 004 and 006 recounted scenarios where they felt professionally obligated to enact their role as public communicators to help promote particular agendas, which did not sit comfortably with their ‘core insider’ status.^10^

Finally, while most interviewees did not suggest line-managing other civil servants affected their independence, one participant described minimizing the management dimensions of their role because they believed, ‘you’re not truly independent if you’re managing people on behalf of the department’ (003).

### Intra-UK variations in the CMO role

Reflecting historical accounts of the CMO role in England,^3^ CMO roles across the UK appear flexible, especially compared to the tightly legislated equivalent role in Canada^4^: ‘Every CMO runs it slightly differently,’ said 003, while 007 stated that ‘there is no two of the roles which are exactly the same in terms of the way that they’re undertaken.’

This flexibility was depicted as advantageous since it enables CMOs to^1^: adapt to external events (useful, since *‘*you can never actually anticipate every single eventuality’ (001)); and^2^ shape the role to better suit preferred working styles (003), or organisational needs (006).

The combination of the role’s flexibility with the devolution of health policy and contextual variations (notably population size; see Supplementary File) mean the CMO role varies across the four UK nations.^2^ This variation (Table 2) shapes the role’s perceived independence. For example, England’s Health Committee in England expressed concerns that the 2010 narrowing of the CMO’s leadership role from medical professions to public health professions might reduce the role’s independence.^20^ An interviewee in England described their counterparts in the devolved nations as less independent because of their greater involvement in policy. Yet, when asked, interviewees from the devolved nations explicitly disagreed. Unpacking this disagreement is challenging since our findings suggest that CMOs and DCMOs working across the four UK nations do not always understand how the role differs. This is important, given the coordination required in public health emergencies^2^ and the requirement for the CMO in England to represent the whole UK at the World Health Assembly (Table 2).

**Table 2:** Intra-UK variations in the CMO role

*Aspect of role*	*UK nation*			
	*England*	*Scotland*	*Wales*	*Northern Ireland*
Professional leadership	In England (the largest UK nation), the CMO’s professional leadership focuses on public health and does not include leadership of the medical professions. This follows changes made to the CMO role in 2010–11, which devolved the original broader duties around medical professional leadership to an NHS Medical Director.^20^ The rationale for this change is set out in evidence given by Dame Sally Davies to the House of Commons Health Committee in 2011.^20^	In the devolved nations, CMOs have a professional leadership role for the medical professions.^21–23^ As the ‘chief professional officer[s] across the medical fraternity’ (008), these duties are vast, including medical education, appraisal, revalidation and best practice guidelines and stakeholder management (001, 007, 008, 010). Serving as intermediaries between the worlds of medicine and government, CMOs in the devolved nations can become, as one participant expressed, ‘a conduit between the profession and the policy that’s developed’ (007). This means that, in the devolved nations, CMOs also have a role in relation to healthcare delivery and services, primarily via the National Health Service (NHS).^21–23^		
Health research and funding leadership	The CMO for England has a more significant role in health research funding than other CMOs and has, at various points over the past decade, also served as the Chief Scientific Advisor to the English Department of Health and Social Care, a role that includes leading the National Institute for Health Research (NIHR) which, with an annual research budget of ~£1billion, funds researchers across the UK. The current CMO for England, Professor Chris Whitty, held this role until April 2021, when it was announced that this part of the role was to become a separate appointment.^24^	In Scotland, the CMO has a role in investing in health research,^25^ but there is also a separate Chief Scientist for Health role, who leads the Chief Scientist Office (CSO) for Scotland, a major funder of health research in Scotland.	In Wales, the CMO is responsible for developing health and care research,^23^ though the budget is more limited and Wales-specific^26^ and there is a separate Chief Scientific Adviser for Health and a Chief Scientific Adviser for Wales.	In Northern Ireland, the CMO has a role in shaping health and social care research funding but there is a there is a separate Chief Scientific Advisor role, which includes leading health and social care research.
Policymaking	England, the CMO has their own office within the Department for Health and Social Care^27^ and the role is consistently described as providing policy ‘advice.’^28^ In interviews, interviewees who had worked in England consistently said that they did not get directly involved in ‘policymaking.’	In Scotland, there is a separate CMO Directorate and, like England, the policy responsibilities are restricted to providing advice.^25^	In Wales and Northern Ireland (the smallest two UK nations), CMOs are located within the Department/Directorate of Health and have a more direct role in policymaking. An interviewee described CMO Wales as ‘head of the policymaking part of public health, not just advisory’ (006) and ‘the senior responsible officer for making sure the legislation was developed through the civil servants’ (006). Similarly, a Northern Irish document states that the responsibilities of the CMO group include the ‘development of policies and standards.’^21^	
International representation	The CMO in England has an international dimension to their role, as an official member of the World Health Assembly. Since the devolved nations do not have equivalent membership, this requires representing the whole UK.	The CMOs of the devolved nations may be invited by the CMO in England to join World Health Assembly meetings, as a guest, or to deputise but they are not formal members. They may also be part of international networks (e.g. CMOs in devolved nations mentioned being part of European CMO networks, WHO policy groups that their respective governments were participating in, and bi-lateral relationships with counterparts in Africa and East Asia).		

### Adapting for public health emergencies

Given the flexibility of UK CMO roles, our data find rapid adaptions during public health emergencies. First, the scientific advisory function intensifies, becoming ‘much, much more significant’ (008), and fast-paced, with a demand for advice at ‘very short notice, and from a range of advisors and networks’ (002).

Second, the frequency and visibility of the public communication function significantly expands, with (especially during COVID-19) intense ‘public and media interest’(007). Interviewee 008 suggested this made it harder to ‘separate’ the spokesperson from the advisor role (echoing experiences in Canada and Australia^8^).

Third, the management dimension of the role may be reduced or delegated. While 009 described struggling with management during a public health emergency, others reported delegating management responsibilities (again echoing Canadian experiences^8^).

Fourth, the advocacy function appeared to be demoted, limiting CMOs’ ability to ‘provide advocacy as universally as we would have wanted to pre-pandemic’ (007). This is important, given the UK COVID-19 Public Inquiry’s concern that inequalities were not adequately considered.^2^

## Concluding discussion

### Main finding of this study

CMO roles are loosely defined in UK legislation but comprise four consistent dimensions across the four nations: scientific policy advisor, public health communicator, line manager and advocate. In public health emergencies, limited statutory parameters for CMOs enable expanding advisory and public communication functions (and associated reductions in management and advocacy). However, this flexibility poses two risks. First, intra-UK variations can make each CMO’s responsibilities and accountabilities unclear. Second, the expansion of the role’s public communication function during public health emergencies (e.g. daily media appearances and regular contributions to official committees) raises questions regarding the role’s independence; can CMOs maintain their role as ‘independent’ advisors in the context of being *perceived* as the public face of government decisions and advice (and is this even desirable)? Broader challenges to the epistemic authority of science and government in the post-pandemic era may mean CMOs’ balancing act becomes harder to maintain.^2^

### What is already known on this topic

Historical analysis already identified that the CMO role in England was flexible, while analysis of the role during the COVID-19 pandemic positions the English CMO as a ‘core insider’, bound by the rules and practices of the civil service.^10^ Recent analysis of the equivalent role in Canada had identified three main role functions: advisory, communication and management.^4^^,^^9^

### What this study adds

CMO roles are flexible across the devolved UK which alongside contextual differences, contributes to intra-UK role variation (Table 2).^2^ Despite this, key role dimensions appear similar across the UK and Canada, although Canadian counterparts also have a legislative mandate to act as independent regulators,^4^^,^^8^^,^^9^ while CMOs in the UK place more emphasis on advocacy. Finally, our analysis confirms the claim that CMOs are ‘core insiders’,^10^ who must reconcile the competing ‘rules of the game’ arising from their dual status as senior civil servants and scientific experts.

### Limitations of this study

In focusing on CMOs’ accounts of the role, we have not considered how other UK scientific advisors perceive it, nor have we explored how CMOs interact with others. Both would be useful bases for future research, given the complexity of the UK’s scientific advisory systems.^2^

## Supplementary Material

Supplementary_file_FINAL_fdae278

## Data Availability

The documentary data sources underlying this article are available in the article and in its online supplementary material, via citations. The interview data used in this article cannot be shared publicly because interviews were provided on the basis that all extracts would be anonymised (so this was the basis of the consent obtained). The unique professional experiences of these participants mean that full transcripts are likely to reveal the identity of the speaker. Aspects of these data, or specific queries about the data, will be shared/responded to on reasonable request to the corresponding author.

## Notes

**K.E. Smith,** Professor

**A. Macintyre,** Honorary Research Fellow

**M. MacAulay,** Research Associate

**P. Fafard,** Professor

## References

[1] MacAulay M, Macintyre AK, Yashadhana A. et al. Under the spotlight: understanding the role of the chief medical officer in a pandemic. J Epidemiol Community Health 2022;76:100–4. 34407995 10.1136/jech-2021-216850 PMC8666819

[2] Hallett H . *UK Covid-19 Inquiry Module 1: The Resilience and Preparedness of the United Kingdom*. https://covid19.public-inquiry.uk/documents/module-1-full-report/. London, UK: His Majesty’s Stationery Office, 2024.

[3] Sheard S, Donaldson L. *'The Nation’s Doctor': The Role of the Chief Medical Officer 1855–1998*. Boca Raton, FL: Radcliffe, 2005.

[4] Fafard P, McNena B, Suszek A. et al. Contested roles of Canada's chief medical officers of health. Can J Public Health 2018;109:585–9. 29981105 10.17269/s41997-018-0080-3 PMC6182332

[5] Jakubowski E, Martin-Moreno JM, McKee M. The governments' doctors: the roles and responsibilities of chief medical officers in the European Union. Clin Med (Lond) 2010;10:560–2. 21413477 10.7861/clinmedicine.10-6-560 PMC4951860

[6] McKee M . The changing role of the chief medical officer for England. BMJ 2017;356:j1545. 28348198 10.1136/bmj.j1545

[7] Keeley P, Taubert M, Wardle E. et al. What makes for a ‘top doc’? An analysis of UK press portrayals of so-called top doctors. BMJ Leader 2024;8:39–42. 10.1136/leader-2022-000735 37423735

[8] MacAulay M, Fafard P, Cassola A. et al. Analysing the ‘follow the science’ rhetoric of government responses to COVID-19. Policy Polit 2023;51:466–85.

[9] Cassola A, Fafard P, Nagi R. et al. Tensions and opportunities in the roles of senior public health officials in Canada: a qualitative study. Health Policy 2022;126:988–95. 36002358 10.1016/j.healthpol.2022.07.009 PMC9296232

[10] Cairney P, Toth F. The politics of COVID-19 experts: comparing winners and losers in Italy and the UK. Policy and Society 2023;42:392–405.

[11] Sargeant J . *Co-Ordination and Divergence: Devolution and Coronavirus*. https://www.instituteforgovernment.org.uk/publication/report/co-ordination-and-divergence-devolution-and-coronavirus. London, UK: Institute for Government, 2020.

[12] Ashton J . The nation’s doctor and the COVID-19 pandemic. J R Soc Med 2021;114:451–3. 34533087 10.1177/01410768211043442 PMC8451008

[13] Nice A . *Explainer: Chief Medical Officer*. https://www.instituteforgovernment.org.uk/article/explainer/chief-medical-officer. London, UK: Institute for Government, 2020.

[14] Gorsky M . Local leadership in public health: the role of the medical officer of health in Britain, 1872-1974. J Epidemiol Community Health 2007;61:468–72. 17496253 10.1136/jech.2006.046326 PMC2465709

[15] Fee E, Brown TM. The public health act of 1848. Bull World Health Organ 2005;83:866–7. 16302044 PMC2626469

[16] Warren MD . *A Chronology of State Medicine, Public Health, Welfare and Related Services in Britain 1066–1999*. https://web.archive.org/web/20150924015045/http://www.fph.org.uk/uploads/r_chronology_of_state_medicine.pdf. London, UK: Faculty of Public Health Medicine of the Royal Colleges of Physicians of the United Kingdom, 2000.

[17] The Health and Personal Social Services (General Medical Services Contracts) (Prescription of Drugs Etc.) (Amendment) Regulations (Northern Ireland) 2010 & 2011, 375 (2010).

[18] UK Government . *Statutory Guidance: The Civil Service Code*. https://www.gov.uk/government/publications/civil-service-code/the-civil-service-code. London: The UK Government, 2015.

[19] Scally G, Jacobson B, Abbasi K. The UK’s public health response to covid-19. BMJ 2020;369:m1932. 32414712 10.1136/bmj.m1932

[20] Health Committee (UK Parliament) . *Health Committee Twelfth Report (Public Health)* https://publications.parliament.uk/pa/cm201012/cmselect/cmhealth/1048/104802.htm. London: House of Commons (UK Parliament), 2011.

[21] Human Resources for the Northern Ireland Civil Service and the Northern Ireland Office . Candidate Information Pack: IRC253842 Senior Medical Officer – Health Protection Department of Health (DoH) https://irecruit-ext.hrconnect.nigov.net/resources/documents/i/r/c/irc253842---cib-v3-final.pdf, 2020.

[22] Scottish Government . CMO Scotland Applicant Pack October 2020. https://applications.work-for-scotland.org/pages/job_search_view.aspx?jobId=17072&JobIndex=12020.

[23] Welsh Government . *Sir Frank Atherton: Chief Medical Officer*. https://www.gov.wales/sir-frank-atherton. Cardiff: The Welsh Government, Undated.

[24] NIHR . New Chief Scientific Adviser and NIHR Lead Announced*.* https://arc-eoe.nihr.ac.uk/news-insights/news-latest/new-chief-scientific-adviser-and-nihr-lead-announced, 2021.

[25] Scottish Government . Chief Medical Officer Directorate. https://www.gov.scot/about/how-government-is-run/directorates/chief-medical-officer/Undated.

[26] Welsh Government . CMO Wales Job Advert 2016, Science & Technology Committee, Oral Evidence: Work of the Chief Medical Officer, HC 779, Tuesday 2 February 2016. https://cymru-wales.tal.net/vx/mobile-0/appcentre-1/brand-2/candidate/so/pm/1/pl/6/opp/1598-Chief-Medical-Officer/en-GB, 2016.

[27] Department of Health and Social Care . *Organogram of Staff Roles and Salaries: DHSC Organogram January 2023*. https://www.data.gov.uk/dataset/04427362-663e-49e0-9103-8bc01dcaa2c7/organogram-of-staff-roles-and-salaries/datafile/e07126e8-e240-453a-a4b7-748d7f355ddc/preview#organogram. England: Department of Health and Social Care, 2023.

[28] UK Government . *Chief Medical Officer and Expert Adviser: Professor Chris Whitty*. https://www.gov.uk/government/people/christopher-whitty#current-roles:. UK Government, Undated.

