# Supplementary material for: Chief medical officers in the United Kingdom: maintaining ‘independence’ inside government
Source: J Public Health (Oxf). 2024 Oct 30;47(1):54–61. doi: 10.1093/pubmed/fdae278 (PMC11879052; doi:10.1093/pubmed/fdae278)
Supplement: Supplementary_file_FINAL_fdae278 [file supplementary_file_final_fdae278.docx]

**Supplementary File**

**Table A: Overview of legislative context surrounding the CMO role in the devolved United Kingdom**

| **Legislation Type** | **Legislation** | **Mentions the CMO? How is the role described?** |
| --- | --- | --- |
| **England** | | |
| **Public Health / Health Care** | Public Health (Control of Diseases) Act 1984^2^ | NO |
|  | Public Health (Infectious Diseases) Regulations 1988^[[1]](#footnote-1)^ | States that ‘a proper officer’ must notify the CMO (for England or Wales) regarding disease outbreaks or if rats are infected with plague. |
|  | Health & Social Care Acts 2012 | NO |
| **Other** | Civil Liability Act 2018 | States that when making regulations using the Act the Lord Chancellor must consult with various public bodies and offices including the CMO of the Department of Health & Social Care and the CMO of the Welsh Government. |
|  | Constitutional Reform Act 2005 | This Act amends the Social Security Act and requires the Judicial Appointments Commission, in the process of the appointment of a medical practitioner to appeals tribunals, to consult with the CMO. |
|  | Abortion Act 1967 | Requires that medical practitioners must notify the CMO of terminations of pregnancy. |
|  | Abortion Regulations 1991 | As above – requires medical practitioner to notify the CMO of terminations. |
|  | Care Act 2014 | Under the section on the Health Research Authority, states that the HR must coordinate with a range of officers and public bodies, including the CMO, to ensure that they coordinate and standardise the way in which health or social care research is regulated. |
|  | Coroners & Justice Act 2009 | In the section on notification of deaths there is a provision which states that the CMO to issue guidance on how medical certificates are to be completed. |
|  | Permitted persons (designated) Order 2006 | States that the CMO is one of the ‘permitted persons’ “*allowing them to disclose information to the Judicial Appointments Commission for the purposes of selection under Part 4 of the Constitutional Reform Act”* |
| **Emergency** | Coronavirus Act 2020^[[2]](#footnote-2)^ | Identifies the CMO (and Deputy CMOs) of the Department of Health and Social Care as those whom the Secretary of State must consult with or take advice from in relation to several provisions of the Act. Does not specify what the CMO must do. |
|  | The Statutory Sick Pay (Coronavirus) (Suspension of Waiting Days and General Amendment) Regulations 2020 | States that the CMO (and Deputy CMOs) can specify/amend additional symptoms of Coronavirus in guidance. |
| **Wales** | | |
| **Public Health / Health Care** | National Health Service (Wales) Act 2016 | NO |
|  | National Health Service Finance (Wales) Act 2014 | NO |
| **Other** | Abortion (Amendment) (Wales) Regulations Act 2008 & 2002 | State that the CMO for Wales must be notified of terminations of pregnancy. |
|  | Private and Voluntary Health Care (Wales) Regulations 2002 | State that the CMO for Wales must be notified of terminations carried out at independent hospitals. |
|  | Wellbeing of Future Generations Act 2015 | The CMO is mentioned in this Act as being a member of the advisory panel which advises the Future Generations Commissioner for Wales. |
| **Emergency** | Coronavirus Act 2020 | See above for England. The Act requires Welsh Ministers to consult with or have regard to the advice of the CMO or Deputy CMOs of the Health and Social Services Directorate before making declarations under the Act. |
|  | Health Protection (Coronavirus Restrictions) (Functions of Local Authorities etc.) (Wales) Regulations 2020 | Requires Welsh Ministers to consult with the Chief Medical Officer for Wales. |
| **Scotland** | | |
| **Public Health / Health Care** | Public Health (Scotland) Act 2008 | NO |
|  | Public Health (Notification of Infectious Diseases) Scotland) Regulations 1988 | States that the chief administrative medical officer must notify the CMO of disease outbreaks. |
|  | NHS Reform (Scotland) Act 2004 | NO |
|  | National Health Service and Community Care Act 1990 | NO |
|  | the National Health Service (Scotland) Act 1978 | NO |
| **Other** | Social Security (Scotland) Act 2018 | States that the CMO should prepare *“guidance that sets out when a progressive disease can reasonably be expected to cause an individual's death for the purpose of determining entitlement to disability assistance.”* |
|  | Social Security Administration and Tribunal membership (Scotland) Act 2020 | Modifies the above Social Security (Scotland) Act 2018 to state that in preparing or revising guidance the CMO must consult *“appropriate healthcare professionals”*. |
|  | Public Records (Scotland) Act 2011 | Notes that the Chief Medical Officer is part of the Scottish Administration |
|  | Freedom of Information (Scotland) Act 2002 | Defines the CMO as one of the *“Non-Ministerial Officer Holders in the Scottish Administration”*. |
|  | Scottish Administration (Offices) Order 1999 | This legislation outlines the non-ministerial offices for the purposes of the Scotland Act 1998. The CMO is included as one of those such offices. |
|  | The Abortion (Scotland) Regulations 1991 | States that “*any practitioner who terminates a pregnancy in Scotland”* must notify the Chief Medical Officer. The Act also has a provision restricting the disclosure of such information provided to the CMO. |
| **Emergency** | Coronavirus Act 2020 | See above for England. The Act requires Scottish Ministers to consult with, or have regard to the advice of, the Chief Medical Officer of the Scottish Administration when issuing and updating guidance or directions. |
| **Northern Ireland** | | |
| **Public Health / Health Care** | Public Health Act (Northern Ireland) 1967 | NO |
|  | Health & Social Care (Reform) Act (Northern Ireland) 2009 | NO |
|  | The Health and Personal Social Services (General Medical Services Contracts) (Prescription of Drugs Etc.) (Amendment) Regulations (Northern Ireland) 2010 & 2011 | In both these regulations the CMO is mentioned as the way in which the Department of Health, Social Services and Public Safety notifies general practitioners and other health professionals that there is an outbreak of influenza in the community (via a letter from the CMO). |
| **Other** | The Prison and Young Offenders Centre (Amendment) Rules (Northern Ireland) 2009 | This amendment replaces the mention of Chief Medical Officer in relation to: Rule 1 (medical examination on reception) – “the chief medical officer” is replaced with *“a registered general practitioner, the health care manager and the Board”.* Rule 29 (death or serious illness of prisoner) – “the chief medical officer” is substituted with “*“the Assistant Director of Prison Health Care”* |
|  | The Social Security (Northern Ireland) Order 1998 | States that the Northern Ireland Judicial Appointments Commission must consult with the Chief Medical Officer, in the process of appointing a medical practitioner to a panel for appeal tribunals. |
| **Emergency** | Coronavirus Act 2020 | See above for England. States that “the Department” must consult with, or have regard to the advice from, the Chief Medical Officer or Deputy Chief Medical Officers of the Department of Health in Northern Ireland. |

**Table B: Public administration analysis of the CMO role across the four countries of the devolved United Kingdom**

| **Country (Population, mid-2020)** | **Professional leadership role** | **Medical leadership role within Government** | **NHS / healthcare role** | **Health research leadership role** | **Scientific Advisory Governance role (during COVID-19)** | **International role** |
| --- | --- | --- | --- | --- | --- | --- |
| **England**  (56,550,000) | Head of the public health profession in England.  *“Provide professional leadership to PHE^[[3]](#footnote-3)^, Directors of Public Health, and other public health professionals^[[4]](#footnote-4)^* | *“Head of profession to medical staff in Government”* ^[[5]](#footnote-5)^ | Does not appear to have a direct role in the operational side of the NHS. | In the past decade the role has, at times, incorporated the Chief Scientific Advisor to the Department of Health & Social Care, which includes leading the National Institute for Health Research (NIHR)^[[6]](#footnote-6)^. As of August 2021, these roles were again separated. | Co-chair of SAGE along with Chief Scientific Advisor to UK Government. | Represents the UK at international meetings and global health institutions (e.g. the WHO)^[[7]](#footnote-7)^ |
| **Scotland**  (5,466,000) | Head of the medical profession in Scotland.  “*the leader of the Medical Profession in Scotland”^[[8]](#footnote-8)^* | In addition to professional leadership role, also required to *“Provide leadership and manage the professional development of all medical staff in the Scottish Government Health and Social Care Directorates”^8^* | Advises on healthcare: “*to oversee the effectiveness of healthcare services in Scotland.”^8^* The CMO for Scotland reports jointly to the Director General for Health and Social Care and the Chief Executive of NHS Scotland. | The CMO is supported by the Chief Scientist for Health, which is a separate role, focused primarily on supporting medical/health research via the ‘Chief Scientist Office for Scotland’ (CSO for Scotland). However, CMO Office reported as having a role in *“investing in research, particularly related to the NHS”*^[[9]](#footnote-9)^ | Member of SAGE during COVID-19 pandemic^[[10]](#footnote-10)^. Member of Scottish Government Advisory Group which reports through the CMO Office but is co-chaired by former and current Chief Scientists for Health. | It is recorded that the CMO for Scotland: *“Be the main point of contact with the CMOs from the other UK countries and senior figures in organisations such as the World Health Organisation, the EU and internationally”^9^* |
| **Wales**  (3,170,000) | Head of the medical profession in Wales^[[11]](#footnote-11)^ | Leading (Acting as ‘Responsible Officer’) for Doctors within the Welsh Government^[[12]](#footnote-12)^ | Incorporates the Medical Director for NHS Wales role^[[13]](#footnote-13)^ and includes “*leading the clinical contribution in Wales to improving the quality of healthcare and patient outcomes”*. so has a role in the operational side of the NHS. | The CMO is recorded as being responsible for *“the development of health and care research in Wales”*^12^ | Not a member of SAGE during COVID-19 pandemic. Tactical Advisory Cell (not chaired by CMO, but by the Chief Scientific Adviser for Health and the Deputy Director for Technology and Digital Data). | Not a clear international dimension to the role from analysis of public administration documents. |
| **Northern Ireland**  (1,896,000) | Interviews suggest CMO is head of the medical profession in Northern Ireland. Documentary evidence indicates that Directors of Public Health are *“professionally accountable to the Chief Medical Officer”^[[14]](#footnote-14)^.* | Public administration documents do not record this. | CMO has responsibility to *“improve the safety and quality of Health and Social Care services through the provision of advice and leadership and the development of policies and standards”*.^[[15]](#footnote-15)^ | There is a separate Chief Scientific Advisor position which includes the role of Director of Health and Social Care Research and Development^[[16]](#footnote-16)^ | Not a member of SAGE during COVID-19 pandemic (Chief Scientific Advisor is a member). No formal scientific advisory group established. | No clear international dimension to the role from analysis of public administration documents. |

**Example Semi-Structured Interview Guide:**

*Preliminary Matters/ Opening Questions*

(Briefly introduce interviewer and scope of the project and re-check ethics and consent form)

1. **We wanted to interview you for this project given your experience of the role of CMO which we understood you took up in [personalise to interviewee]. As an opening question, I’d like to ask if you can recall what you thought this role would entail when you were first appointed and how they ended up comparing with the day-to-day reality?**
2. **The CMO role is clearly multi-faceted. What do you see as the core functions of the role?**
   - In your experience, do any of these roles conflict with one another, and how do you manage this?
   - [Probe - If not explicitly covered above] – Our assessment suggests that the CMO role can involve both medical and public health dimensions. Would you agree, and if so, how do you approach and balance these different aspects of the role?
   - How do you feel the CMO role has adapted in the context of the need to support the policy response to COVID-19?
   - [Probe – if not explicitly covered above] Public health traditionally involves three domains – health protection, health services and health promotion. What was your approach to these three core areas? Were these aspects ever competing, and if so, how did you manage this?
   - [Probe – if not explicitly covered above] Health promotion can include tackling the social determinants of health and health inequalities. To what extent did you feel this was part of the CMO role?
   - [Probe - What do you see were the main challenges/barriers to this?

*Structural and Contextual Features*

1. **The CMO role obviously involves interacting with other CMOs in the UK. Could you tell us a bit about how you worked together and exchanged ideas?**

1. **Could you tell us a little bit about your reporting relationships and which ministers and other senior officials you commonly worked with in your role?**
2. **To what extent do you feel the CMO role involves or requires some independence from government?**
3. **Thinking about the structural, legislative and historical precedents that have shaped the CMO role in [this nation], what factors do you feel enhance the ability of this role to provide public health leadership during the pandemic? And are there any aspects of how the role has evolved in [this nation] that you felt brought challenges to undertaking the role in the unusual context of a global pandemic?**

*Advice*

1. **In exercising the CMO role of providing advice to the government, how would you categorise the types of issues that you were called upon to give advice on?**
   - How do you manage/approach these different areas?
   - Are these different issues ever competing and if so, in what ways?

- With whom, if anyone, did you share the responsibility for providing advice on public health matters? How was this shared responsibility managed?

1. **How would you describe the CMO’s advisory relationship to Ministers and other senior government officials?**
   - What kinds of factors shape CMO access to ministers and officials and does this vary over time? Did any changes relate more to structures or individuals and their professional relationships?
   - To what extent did you provide advice directly to Ministers or senior officials?

*Communications/Advocacy*

1. **Would you say that CMOs speak on behalf of the government, and if so, is this a major or minor part of the role?**
   - What informed whether you accompany ministers when they are making a major announcements relating to public health?
2. **Advocacy is increasingly depicted as a core competency in public health training. To what extent did you see your role as involving advocacy and, if so, how did you approach it?**
   - Advocacy can sometimes involve being critical of, or trying to change, existing policy. In situations where you have felt that existing policy requires change, how did you approach this?
     1. To what extent did you feel it was possible, in the context of this role, to be critical of existing policy, either publicly or privately?
   - The World Health Organization and many others take the position that to address the social determinants of health requires action on policy areas outside the remit of health policy. How did you approach this issue and how would you like to see it taken forward?
3. **Could you tell us a bit about your role in issuing reports to the public on public health issues?**
   - What was the process like for developing and signing off a CMO report – who is involved in the drafting stages, who is required to sign off, etc?
   - How did you feel about this?
   - We are finding that, in some jurisdictions but not others, the CMO has the authority, under legislation, to issue reports to the public on public health issues. What is your understanding of whether such authority exists in legislation in different parts of the UK? What are the advantages and disadvantages of this?
   - Are you aware of any examples in which CMO reports have been in tension with other parts of the government? If so, could you tell me a bit about how this was managed?

*Management*

1. **To what extent would you say management is part of the CMO role in [this nation]?**
2. **Does the CMO here exercise any oversight over national or local public health officials or programmes and, if so, how does this work?**
   - How much of this was the result of how public health is structured and how much of it was more informal?
3. **To what extent, if any, would you say the pandemic impacted on the management responsibilities of the CMO role?**

*Concluding questions*

1. **One of the most interesting things we have observed about the CMO role in the context of the pandemic, and something most interviewees so far have commented on, is how prominent and visible the role has become. Do you feel this high public visibility helps or hinders CMOs during a pandemic?**
2. **Looking back on your experiences, if you could provide one piece of advice to anyone taking on the CMO role anew, what would it be?**
3. **Is there anything you would like to add that would help us better understand your experiences as CMO?**
   - Based on your experiences, what do you feel is the best way to design the role of CMO to ensure maximum effectiveness
   - [Alternate] ‘how, if at all, would you alter the role to ensure maximum effectiveness?’

1. The Public Health (Control of Diseases Act (1984) and The Public Health (Infectious Disease) Regulations (1988) apply in both England and Wales [↑](#footnote-ref-1)
2. The Coronavirus Act (2020) covers England, Wales, Scotland and Northern Ireland. There are different provisions for each country, but the framing of the CMO role is similar across all four jurisdictions. Secondary legislation which includes similar statements on the CMO role to those in the Act include: The Health Protection (Coronavirus, Restrictions) (No.2) (England) Regulations 2020; The Health Protection (Coronavirus Restrictions) No.3) Regulations 2020; The Health Protection (Coronavirus, Public Health Information for Passengers Travelling to England) Regulations 2020; The Health Protection (Coronavirus, Restrictions) (Directions by Local Authorities) (Scotland) Regulations 2020 [↑](#footnote-ref-2)
3. PHE – Public Health England [↑](#footnote-ref-3)
4. CMO Job Advert England, 2019 [↑](#footnote-ref-4)
5. CMO England Candidate Pack, 2019 [↑](#footnote-ref-5)
6. Oral evidence to the House of Commons Science & Technology Committee, February 2016 [↑](#footnote-ref-6)
7. Jakubowski et al 2010; CMO England Candidate Pack; McKee 2017 [↑](#footnote-ref-7)
8. CMO Scotland Applicant Pack, October 2020 [↑](#footnote-ref-8)
9. <https://www.gov.scot/about/how-government-is-run/directorates/chief-medical-officer/> [↑](#footnote-ref-9)
10. Sargeant, J. (2020) *Co-ordination and divergence. Devolution and coronavirus*, Institute for Government https://www.instituteforgovernment.org.uk/sites/default/files/publications/coordination-divergence-devolution-coronavirus.pdf [↑](#footnote-ref-10)
11. <https://gov.wales/dr-frank-atherton> [↑](#footnote-ref-11)
12. CMO for Wales Job Advert, 2015/16 [↑](#footnote-ref-12)
13. CMO Wales Job Advert 2016 <https://cymru-wales.tal.net/vx/mobile-0/appcentre-1/brand-2/candidate/so/pm/1/pl/6/opp/1598-Chief-Medical-Officer/en-GB> [↑](#footnote-ref-13)
14. Specimen Job Description, Director of Public Health – Northern Ireland, Faculty of Public Health, <https://www.fph.org.uk/media/1923/director-of-public-health-northern-ireland-job-description-2018.doc> [↑](#footnote-ref-14)
15. Human Resources for the Northern Ireland Civil Service and the Northern Ireland Office (2020). Candidate Information Pack: IRC253842 Senior Medical Officer – Health Protection Department of Health (DoH) URL: <https://www.health-ni.gov.uk/dhssps-chief-medical-officer>. [↑](#footnote-ref-15)
16. <https://www.health-ni.gov.uk/news/new-chief-scientific-advisor-appointed> [↑](#footnote-ref-16)
